# Material design for oral insulin delivery

**DOI:** 10.1007/s44258-023-00006-y

**Published:** 2023-07-11

**Authors:** Kangfan Ji, Yuejun Yao, Xinwei Wei, Wei Liu, Juan Zhang, Yun Liu, Yang Zhang, Jinqiang Wang, Zhen Gu

**Affiliations:** 1 Key Laboratory of Advanced Drug Delivery Systems of Zhejiang Province, College of Pharmaceutical Sciences, Zhejiang University, Hangzhou, 310058 China; 2 Jinhua Institute of Zhejiang University, Jinhua, 321299 China; 3 National Key Laboratory of Advanced Drug Delivery and Release Systems, Zhejiang University, 310058 Hangzhou, China; 4 Second Affiliated Hospital, Zhejiang University School of Medicine, Hangzhou, 310009 China; 5 Department of General Surgery, School of Medicine, Sir Run Run Shaw Hospital, Zhejiang University, Hangzhou, 310016 China; 6 Zhejiang Laboratory of Systems & Precision Medicine, Zhejiang University Medical Center, Hangzhou, 311121 China; 7 MOE Key Laboratory of Macromolecular Synthesis and Functionalization, Department of Polymer Science and Engineering, Zhejiang University, Hangzhou, 310027 China

**Keywords:** Drug delivery, Oral administration, Material design, Insulin delivery, Glucose-responsive

## Abstract

Frequent insulin injections remain the primary method for controlling the blood glucose level of individuals with diabetes mellitus but are associated with low compliance. Accordingly, oral administration has been identified as a highly desirable alternative due to its non-invasive nature. However, the harsh gastrointestinal environment and physical intestinal barriers pose significant challenges to achieving optimal pharmacological bioavailability of insulin. As a result, researchers have developed a range of materials to improve the efficiency of oral insulin delivery over the past few decades. In this review, we summarize the latest advances in material design that aim to enhance insulin protection, permeability, and glucose-responsive release. We also explore the opportunities and challenges of using these materials for oral insulin delivery.

## Highlights

- Acid- and enzyme-resistant materials are designed to protect insulin from the harsh gastrointestinal environment.
- Zwitterionic materials, cell-penetrating peptides or ligands modified materials improve intestinal permeability.
- Glucose-responsive moiety achieves on-demand release of insulin and desirable diabetes management.

## Introduction

Diabetes mellitus affects over 536 million people’s lives worldwide [1]. To date, subcutaneous insulin injections remain the primary treatment method for type 1 and advanced type 2 diabetes [2]. However, repeated subcutaneous injections can cause side effects and discomfort. Non-invasive insulin delivery is urgently demanded as they offer the advantages of convenience, good compliance and safety [3]. In addition, subcutaneous insulin injection can lead to peripheral hyperinsulinemia and a high risk of hypoglycemia. As a comparison, oral administration can mimic endogenous insulin distribution with a portal-to-peripheral gradient and ensure normoglycemia with a low risk of hypoglycemia [4].

Nevertheless, insulin, as a peptide, cannot be absorbed efficiently by the gastrointestinal tract because of several biological barriers [5]. First, the harsh environment of the gastrointestinal tract, including the extreme low pH value in the stomach and numerous proteases, destroys insulin readily [6]. Second, the mucus layer consisted of highly glycosylated and negatively charged mucin has a mesh-like 3D structure, protecting the enterocytes from direct contact with pathogens and biomolecules. The small pore size of mucin, which averages between 10 and 500 nm, combined with its various physical interactions, impedes insulin diffusion [7]. Third, the epithelium layer further blocks insulin from entering the systemic circulation [8]. The transcellular and paracellular pathways of crossing the epithelium layer are impeded by multiple factors, including the lack of uptake pathways, lysosomal degradation, and tight junctions’ spatial restriction [9]. Overall, the extremely acidic environment, enzymatic degradation, mucus hindrance, and a tight epithelium layer pose challenges in elevating the bioavailability of oral insulin delivery [10]. After being transported into the blood, the oral insulin delivery systems are required to release insulin in a glucose-responsive manner to enhance the management of blood glucose levels. Therefore, many acid-resistant, surface-functionalized, and glucose-responsive materials have been designed to overcome these challenges in recent years. The carriers derived from these materials can assist insulin in remaining stable in the gastrointestinal tract and reaching the bloodstream to play a hypoglycemic role [11].

In this review, we highlight the recently-developed paradigm of material design for overcoming the aforementioned barriers to realize elevated bioavailability of insulin and improved blood glucose control. Furthermore, we briefly address the opportunities and challenges associated with the clinical translation and development of these new materials.

## Recent progress in material design for efficient oral insulin delivery

**Fig. 1:**
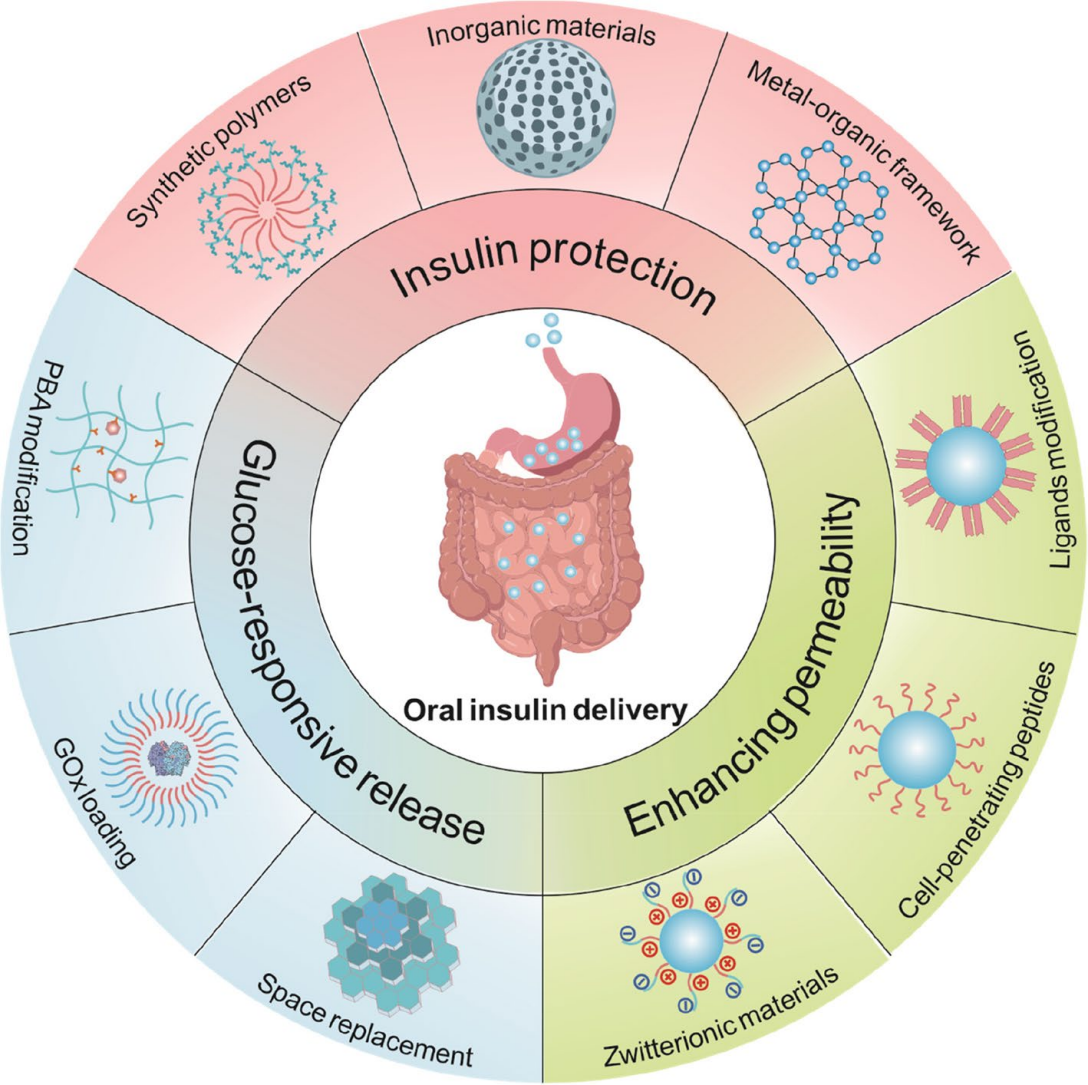
Representative strategies of enhanced oral insulin delivery and associated materials design. PBA, phenylboronic acid; GOx, glucose oxidase

Efficient oral insulin delivery is crucial to normalize blood glucose levels in individuals with diabetes while minimizing potential side effects. Advancements in material science have led to the development of acid- and enzyme-resistant carriers that can protect insulin within the gastrointestinal tract. In addition, materials that contain penetrative and glucose-responsive elements have also been designed and integrated into carriers to enhance bioavailability and treatment efficacy. This section highlights various material-design strategies and related samples (Fig. 1) [9–11].

### Insulin protection

Insulin can be degraded in the presence of protease in the gastrointestinal tract; therefore, preserving the structural integrity of insulin before systemic circulation is a prerequisite for achieving insulin’s therapeutic effect. Thus, insulin requires to be encapsulated in proper vehicles or engineered to resist protease-mediated degradation [12]. Recent advancements in material science have led to the design of various material types, such as synthetic polymers, inorganic materials, and metal–organic frameworks (MOFs), aiming at preparing carriers to improve the stability of insulin within the gastrointestinal environment [11]. The design methodology and properties of each type of material will be introduced below.

**Fig. 2:**
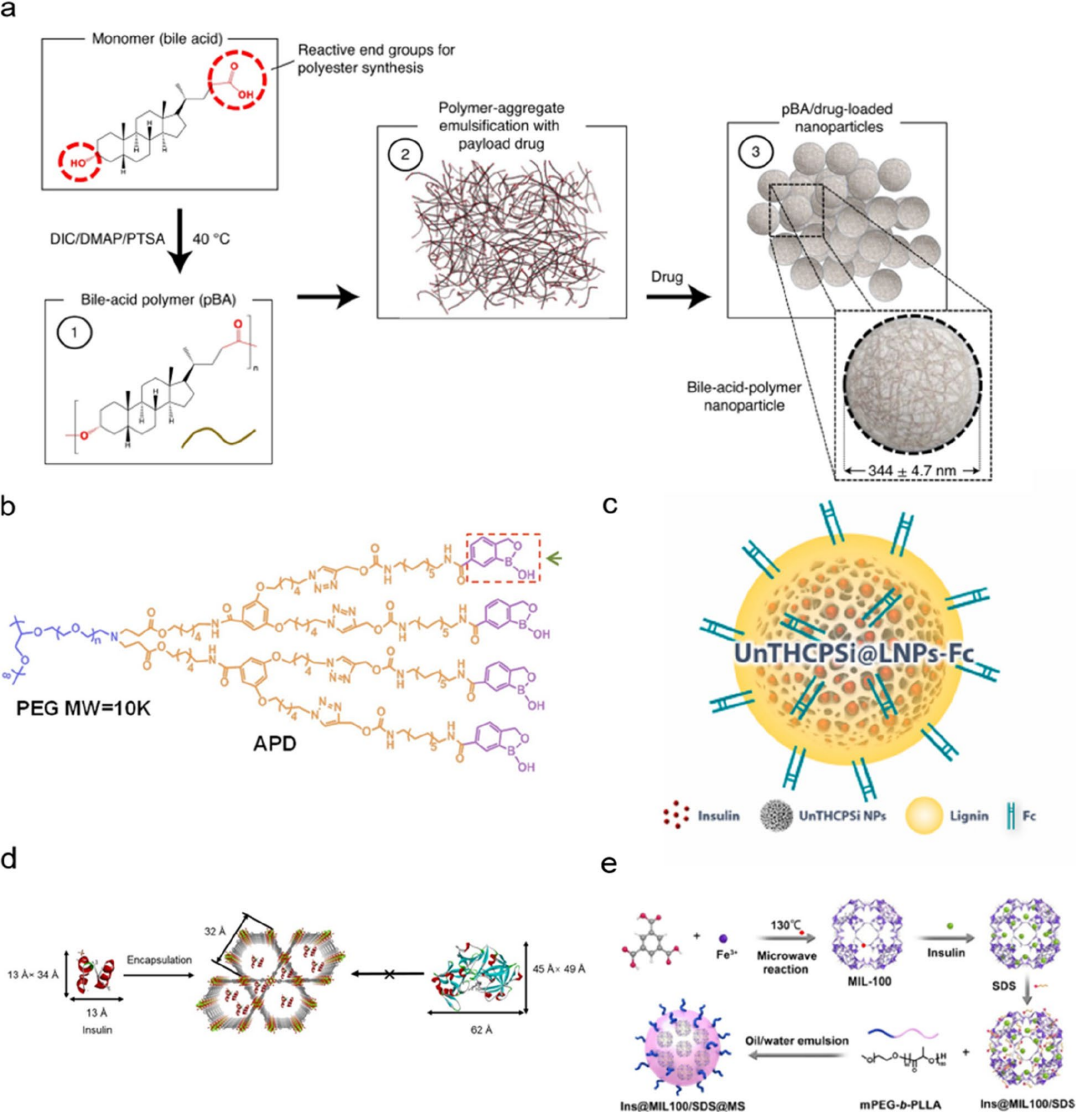
**a** Schematic of bile-acid-polymer nanoparticle used for oral insulin delivery. Adapted with permission from ref [13]. Copyright 2021, The Author(s), under exclusive licence to Springer Nature Limited. **b** Chemical structure of pH-responsive amphiphilic dendrimers designed for oral insulin delivery. PEG, polyethylene glycol; APD, amphiphilic dendrimers. Adapted with permission from ref [14]. Copyright 2019 Elsevier B.V. All rights reserved. **c** Schematic of lignin-encapsulated porous silicon nanoparticle modified with fragment crystallizable (Fc) designed for loading insulin. UnTHCPSi NPs, undecylenic acid modified thermally hydrocarbonized porous silicon nanoparticles. Adapted with permission from ref [20]. Copyright 2021 The Authors. Publishing services by Elsevier B.V. on behalf of KeAi Communications Co. Ltd. **d**, **e** Design of metal–organic framework (MOF) for oral insulin delivery. MIL-100, iron-based metal–organic framework nanoparticle; SDS, sodium dodecyl sulfate; PLLA, poly (L-lactide); Ins, insulin; MS, microspheres. Adapted with permission from ref [22, 24]. Copyright 2018 and 2020, American Chemical Society

The extreme acidic and enzyme-rich environment of gastrointestinal tract predisposes insulin to denaturation. As a result, synthetic polymers that can minimize premature insulin release or reduce enzyme-insulin interaction can maximally retain insulin’s structure integrity and physiological activity. Regarding the capability of acidic groups to be protonated in an acidic environment and become poorly soluble in an aqueous solution, various acid-group-derived materials have been designed and studied. For example, Fahmy and co-workers have prepared polymerized ursodeoxycholic acid for encapsulating insulin (Fig. 2a). Under acidic conditions, this material protonated and became hydrophobic, resulting in reduced penetration of gastrointestinal fluid into particles, inhibiting insulin release and thus protecting insulin from degradation. In the stomach milieu, this formulation has shown minimal insulin leakage [13]. Similarly, Feng and co-workers have reported on the development of a benzoboroxole-containing multi-armed polyethylene glycol (PEG) amphiphilic dendrimer, which became hydrophobic in strongly acidic conditions. When insulin was loaded in this material-based carrier, less than 1% premature insulin release was observed in acidic conditions within four hours (Fig. 2b) [14]. The hyaluronic acid-modified core–shell structure was also reported to maintain insulin stability and showed less than 10% leakage of insulin within 12 h despite strong acidic conditions [15]. In addition, Sun and co-workers have prepared materials based on hydroxypropyl-*β*-cyclodextrin that has a relatively hydrophobic central cavity and a hydrophilic outer surface. Its host–guest interactions with insulin serve to hinder the degradation of insulin by enzymes [16].

Inorganic materials, including gold and silicon-based materials, can resist the acidic condition and enzymatic degradation in the gastrointestinal tract, protecting the encapsulated insulin [17]. Silicon-based materials have high physiochemical stability, biocompatibility, and tunability [18]. Brayden and co-workers have prepared silica-coated nanoparticles loaded with insulin, zinc, and *L*-arginine, enabling a slow release of insulin in the acidic environment [19]. In another research, lignin-encapsulated porous silicon nanoparticles were engineered to deliver insulin (Fig. 2c) [20]. The nanoparticles with or without lignin encapsulation both showed negligible insulin release at pH 1.2 and 6.5, demonstrating excellent insulin-protective capability [20].

MOFs are a class of hybrid crystalline materials consisting of organic ligands and metal ions based on reticular chemistry [21]. MOFs can be designed to have porous structures for loading and protecting insulin from harsh gastrointestinal environments. Farha and co-workers have loaded insulin within NU-1000, an acid-stable Zr_6_-based porous MOF. The NU-1000 was designed with a pore size of approximately 3 nm and was suitable for loading insulin (~ 2 nm in monomeric form) and excluding pepsin (~ 6 nm) (Fig. 2d) [22]. Hence, the insulin-loaded UN-1000 demonstrated minimal release of insulin and strong insulin protection in the simulated stomach acid solution [22]. Similarly, Tian and co-workers have reported an insulin delivery system based on UiO-68-NH_2_ with a diameter of ~ 2.6 nm [23]. Insulin-loaded UiO-68-NH_2_ released more than 90% of insulin in PBS (pH 7.4) and less than 20% in simulated gastric and intestinal fluids, demonstrating an excellent protective effect of MOFs for insulin [23]. Besides, the release of insulin under varied pH conditions can also be controlled. Chen and co-workers have designed a nanocomposite vehicle based on iron-based MOF nanoparticles (MIL-100) with a 2.6 nm pore size (Fig. 2e) [24]. At pH 7.4, the particle size of insulin tetramer was 2.5 ± 0.3 nm [25]. However, the size of insulin aggregates increased rapidly as the pH approached the isoelectric point. Consequently, the release of insulin was almost inhibited in intestinal fluid (pH 5.4) and accelerated dramatically in the blood (pH 7.4) [24].

In addition to materials encapsulation, engineering insulin with amino acid alterations has also shown a potential to protect insulin from proteolytic degradation [26]. The deletion of B27 Thr and substitutions of amino acid at A14 Tyr and B25 Phe were proven to increase the stability of human insulin in pepsin by 38.5-fold. The alterations of amino acid and lipidation also reduced the insulin receptor affinity, contributing to a longer pharmacokinetic profile. Remarkably, with the above strategies, the insulin analogue OI338 has been tested in Phase 2a clinical trial with a similar hypoglycemic effect as insulin glargine [27].

In general, acid-resistant synthetic polymers and inorganic materials can be used for the oral delivery of insulin to maintain the integrity of the carrier and minimize the premature release of insulin in the stomach. The strong interaction between insulin and these materials can prevent the premature release of insulin and protect it from degradation caused by protease-mediated hydrolysis, thereby preserving the activity of insulin. Moreover, strategic modifications of insulin at specific sites can significantly enhance its resistance to protease degradation. Ensuring the passage of insulin through the stomach with the help of carriers is a crucial requirement for efficient absorption.

### Permeability enhancement

The limited permeation through the mucus and intestinal epithelium layers is another challenge for oral insulin delivery. The mucus is a viscous liquid mainly composed of glycosylated and lipid-modified mucin, with negative charges and hydrophobic properties [7]. Therefore, cationic and hydrophobic materials can be easily trapped by the mucus, while neutrally charged and hydrophilic materials, such as zwitterionic and polyethylene glycol-modified materials, can efficiently penetrate mucus [28]. In addition, the size of particles is also a key factor affecting mucus penetration. The large size always impedes mucus permeation due to the mucus’ mesh-like structure with pore sizes in the range of 10 to 500 nm [29]. After getting through the mucus layer, carriers that are cationic and lipophilic can be easily internalized and transported by the intestinal epithelium, despite the contrasting properties required for successful mucus penetration [30]. Hence, designing materials with proper surface properties is crucial to penetrate the mucus layer, pass through the epithelial layer, and deliver insulin into blood circulation. As such, zwitterionic materials, cell-penetrating peptides, and intestinal receptor-specific ligands are incorporated into oral delivery systems to work together to overcome these barriers. In this section, we will focus on the design of materials used to enhance insulin permeability in recent years.

**Fig. 3:**
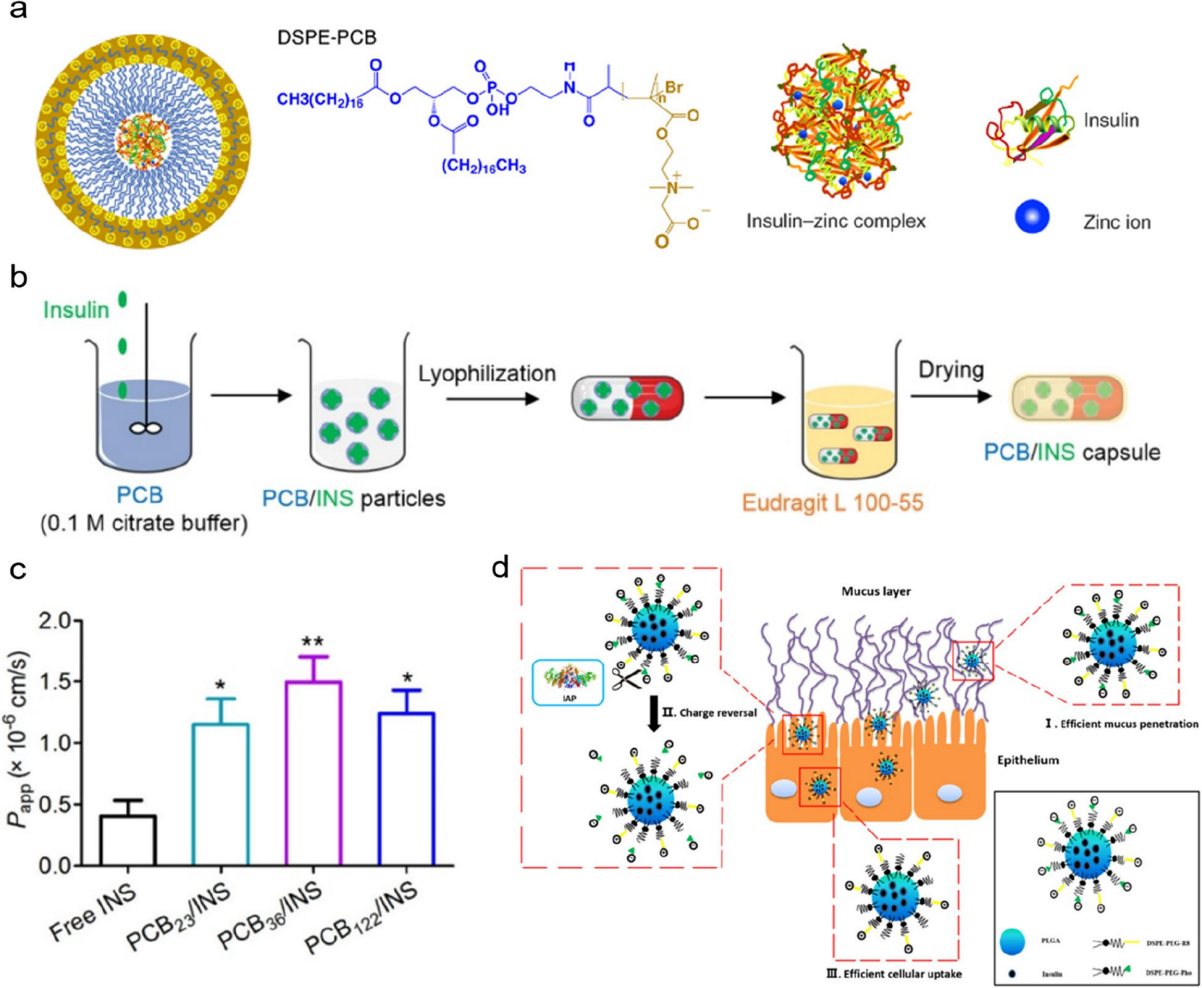
**a** Schematic of zwitterionic micelles for oral insulin delivery. DSPE, 1,2-distearoyl-sn-glycero-3-phosphoethanolamine; PCB, polycarboxybetaine. Adapted with permission from ref [33]. Copyright 2020, The Author(s), under exclusive license to Springer Nature Limited. **b** Fabrication of zwitterionic particles and enteric capsules for oral insulin delivery. **c** The enhanced epithelium uptake of zwitterionic particles loaded with insulin. Adapted with permission from ref [34]. **d** Schematic of zwitterionic nanoparticles modified with the opposite charge for enhancing oral insulin delivery. IAP, intestinal alkaline phosphatase; PLGA, poly (lactic-co-glycolic acid); R8, octa-arginine; Pho, phosphoserine. Adapted with permission from ref [36]. Copyright 2018, American Chemical Society

Zwitterionic materials contain abundant pairs of oppositely charged groups, thus obtaining a neutral charge and robust hydration effect [31]. The zwitterionic materials are reported to penetrate mucus easily due to negligible repulsion or attraction by the negatively-charged mucin [32]. Cao and co-workers have prepared an amphiphilic material composed of 1,2-distearoyl-*sn*-glycero-3-phosphoethanolamine (DSPE) and zwitterionic polycarboxybetaine (PCB) (Fig. 3a) [33]. This material was capable of forming insulin-loaded zwitterionic micelles of about 25 nm diameter in the aqueous phase and had a very low critical micelle concentration, below 10^-6^ mM. The zwitterionic micelles displayed excellent mucus permeability, about 12 times larger than PEG-surfaced particles, and enhanced epithelium uptake mediated by the proton-assisted amino acid transporter 1, achieving 42.6% bioavailability of insulin in diabetic rats [33]. In another research, PCB was used to load insulin directly by electrostatic interaction. The PCB/insulin nanoparticles enabled rapid mucus penetration and enhanced epithelium transport, contributing to a high bioavailability of about 27.0% (Fig. 3b, c) [34]. In addition to PCB, Huang and co-workers have reported an insulin delivery system based on dilauroylphosphatidylcholine with a hydrophilic zwitterionic phosphorylcholine headgroup, the carrier based on which was validated for good mucus penetrating ability and enhanced cellular uptake (4.5-fold) compared to PEGylated nanoparticles [35]. In alternative to the direct modification of zwitterionic materials, researchers also modified the surface of nanoparticles with equal amounts of positively and negatively charged fractions to enhance oral insulin delivery. Huang and co-workers have prepared zwitterionic poly (lactic-co-glycolic acid) (PLGA) nanoparticles by coating the cationic octa-arginine (R8) peptide and specific anionic phosphoserine. The virus-like surface achieved enhanced mucus penetration. Subsequently, the anionic phosphoserine was hydrolyzed as catalyzed by phosphatase, making its surface positively charged and thus more easily transported by epithelial cells (Fig. 3d) [36].

**Fig. 4:**
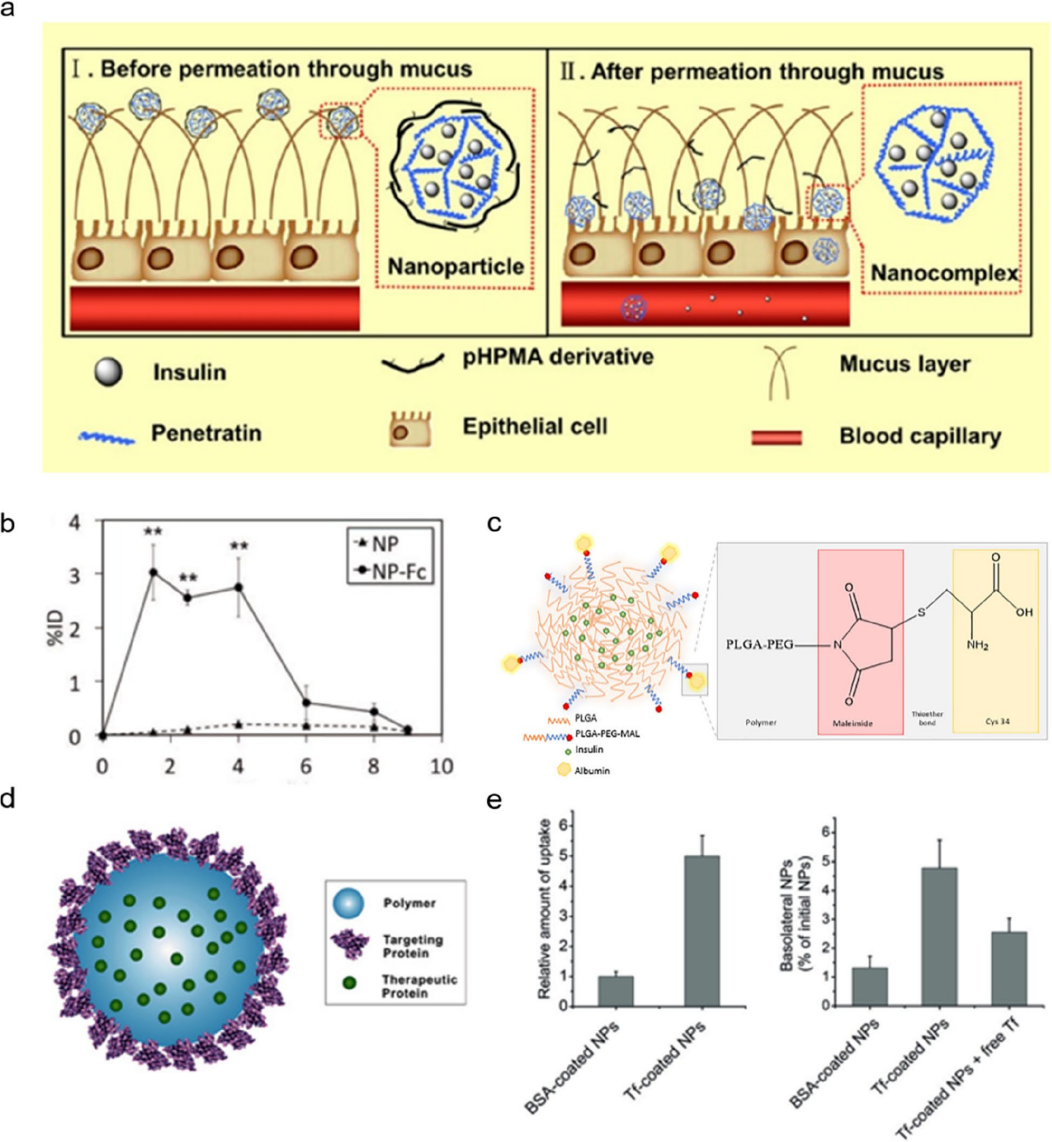
**a** Schematic of nanoparticles with nanocomplex core and N-(2-hydroxypropyl) methacrylamide copolymer derivative shell for enhancing oral insulin delivery. pHPMA, N-(2-hydroxypropyl) methacrylamide copolymer. Adapted with permission from ref [41]. Copyright 2015, American Chemical Society. **b** Significant increase of insulin absorption after Fc modification. Adapted with permission from ref [44]. Copyright 2013, The American Association for the Advancement of Science. **c** The design of albumin-modified nanoparticles for oral insulin delivery. Mal, maleimide. Adapted with permission from ref [46]. Copyright 2020 Elsevier B.V. All rights reserved. **d** Schematic representation of transferrin (Tf)- coated polymer nanoparticles. **e** Enhanced cellular uptake and transepithelial transport of Tf-coated nanoparticles. BSA, bovine serum albumin. Adapted with permission from ref [47]. Copyright 2016 WILEY‐VCH Verlag GmbH & Co. KGaA, Weinheim

In addition, modifying materials with cell-penetrating peptides (CPPs) can enhance the internalization by epithelial cells and the consequent transportation efficiency through the intestinal epithelium. CPPs are amphiphilic and positively charged short peptides that can transport cargo into cells without requiring receptors and energy [37]. Arginine, a semi-essential amino acid, is a major component of many CPPs. Zhang and co-workers modified octaarginine (R8) on the carboxymethyl-*β*-cyclodextrin for enhanced permeability. A three-fold enhanced transport efficiency on Caco-2 cells was observed compared with that of the carrier without R8 modification [38]. Alonso and co-workers conjugated R8 with cholesterol to form a complex with insulin, and then the complex was enveloped by the protecting polymer poly (glutamic acid)-poly (ethylene glycol) [39]. This system showed a good cellular uptake of insulin (47.59 ± 5.79%) but little transportation (2%). In addition to R8, penetratin is another promising CPPs [40]. Researchers engineered nanoparticles with core–shell structures. The core complex was composed of insulin and penetratin. The exterior was reversibly coated with a hydrophilic *N*-(2-hydroxypropyl) methacrylamide copolymer (pHPMA) derivatives (Fig. 4a) [41]. This nanoparticle exhibited good mucus penetration owing to the pHPMA coating and a high cellular uptake mediated by the penetratin. As the nanoparticle passed through the mucus, the pHPMA shells gradually dissociated, contributing a 20-fold higher absorption on the mucus-secreting cellular model than free insulin [41].

Moreover, ligand-mediated insulin transport is also a conspicuous strategy for enhancing carriers’ cellular uptake by intestinal epithelium [42]. Among the varied used ligands, fragment crystallizable (Fc) modification is one of the most used approaches in recent years. The Fc receptor could bind and transport immunoglobulin G in a pH-dependent manner, mediating the translocation across the intestinal mucosal barrier [43]. Based on this, Farokhzad and co-workers modified Fc on the poly (lactic acid)-*b*-poly (ethylene glycol), and this insulin delivery system showed an over 10-times absorption efficiency than nanoparticles without Fc in mice (Fig. 4b) [44]. Subsequently, many researchers modified Fc on the surface of materials to enhance intestinal uptake [15, 17, 45]. In addition to Fc, albumin has also been utilized to improve binding to the Fc receptor. Sarmento and co-workers conjugated albumin and PLGA-PEG to form insulin-loaded nanoparticles, mediating the transport of insulin across the epithelium (Fig. 4c) [46]. Besides, transferrin (Tf) is also a widely-used ligand for oral insulin delivery [24, 47]. For instance, Tf was coated on polymeric nanoparticles by self-assembly to enhance cellular uptake and transepithelial transportation (Figs. 4d, e) [47]. Despite proteins, other ligands such as folate [48], biotin [49], Vitamin B12 [50] and peptides [4, 51] could also be used to modify materials and enhance permeability.

Overall, the utilization of hydrophilic material-coated carriers with sizes smaller than 200 nm have shown promising results by minimizing interaction with the mucus mesh and facilitating smooth penetration through the mucus layer. Additionally, the incorporation of cell-penetrating peptides and ligands that target epithelial cells enhances the uptake of nanocarriers by the epithelium, thereby promoting efficient transepithelial transportation. Among the various materials investigated, one standout candidate is PCB. PCB possesses the desired characteristics of hydrophilicity, neutrality in charge, and a strong affinity for epithelial cells. The simultaneous fulfillment of these criteria makes PCB highly desirable for achieving optimal absorption efficiency.

### Glucose-responsive release

**Fig. 5:**
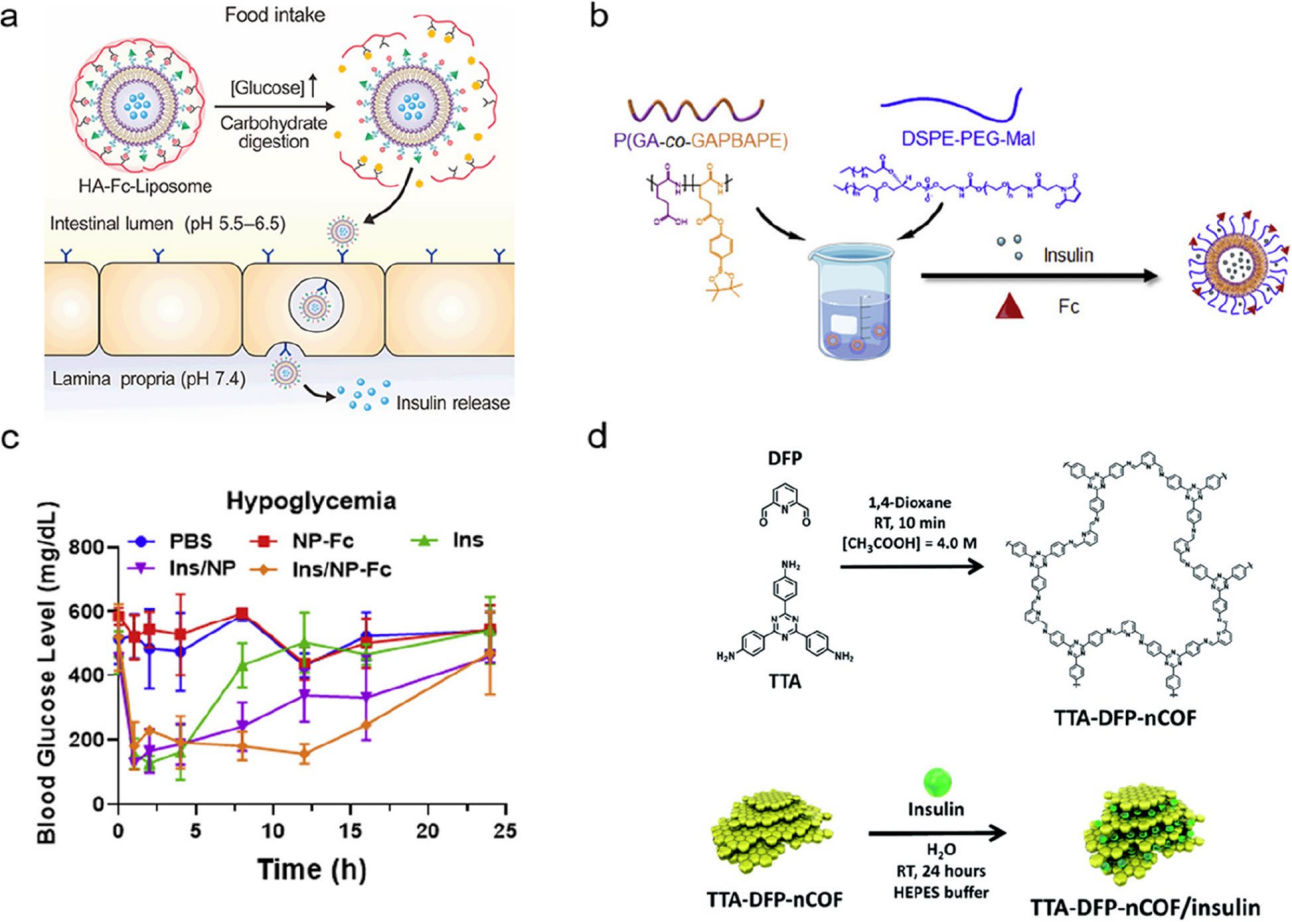
**a** Schematic representation of nanoparticles with glucose-responsive polymer and Fc modification. HA, hyaluronic acid shell. Adapted with permission from ref [15]. Copyright © 2018, Tsinghua University Press and Springer-Verlag GmbH Germany, part of Springer Nature. **b** Schematic of glucose-responsive liposomes and Fc induced cellular uptake for oral insulin delivery. **c** Prolonged hypoglycemia time of mice treated with glucose-responsive nanoparticles. Adapted with permission from ref [45]. Copyright 2021 Elsevier Inc. **d** Chemical structures and schematic illustration of the imine-linked-covalent organic framework for oral glucose-responsive insulin delivery. COF, imine-linked-covalent organic framework; DFP, 2,6-diformylpyridine; TTA, 4,4′,4′′-(1,3,5-triazine-2,4,6-triyl) trianiline. Reproduced from ref [61]. With permission from the Royal Society of Chemistry

Glucose-responsive release of insulin is an efficient way for patients to overcome the risk of hypoglycemia and achieve desirable diabetes management [52–55]. Therefore, several insulin delivery systems based on glucose-binding molecules, glucose oxidase (GOx), and phenylboronic acid (PBA) have been developed to sense the variation in glucose concentration [56–60]. Hence, researchers have also designed glucose-responsive materials for oral insulin delivery to better control blood glucose levels. For instance, Gu and co-workers have engineered a liposome with a core–shell structure [15]. The PBA conjugated hyaluronic acid shell could reversely bind with the catechol and detach rapidly when the postprandial intestinal glucose increased (Fig. 5a). Subsequently, the exposed Fc could facilitate insulin uptake and reduce the postprandial glucose fluctuations in diabetic mice [15]. Similarly, researchers have prepared insulin loaded nanoparticles based on DSPE-PEG-Mal and a glucose-responsive polymer poly (L-glutamic acid-*co*-L-glutamyl phenylboronic acid pinacol ester). The nanoparticles were further modified with Fc (Fig. 5b). The phenylboronic acid pinacol ester could bind with glucose, leading to increased hydrophilicity and negative charge density and realizing the glucose-responsive release of insulin (Fig. 5c) [45]. In addition, GOx and insulin could be loaded into the H_2_O_2_ sensitive polymer methoxypolyethylene glycol-polymethionine. Upon polymer oxidation under hyperglycemic conditions, insulin was released and regulated high blood glucose levels. Also, the blood glucose level could be maintained even after an oral glucose administration [4]. Trabolsi and co-workers have reported a glucose-responsive material based on an imine-linked-covalent organic framework (nCOF). The nCOF consisted of stacked porous nanosheets with a height of 7 nm and a pores size of 1.7 nm. Insulin (~ 2.5 nm) can only be loaded between the nanosheets, while glucose (~ 0.8 nm) can be loaded inside the nCOF pores. At normoglycemic concentrations, the nCOF pores are filled. Under hyperglycemia, glucose is forced to diffuse through the pores and replace the space between nanosheets, therefore triggering the release of insulin (Fig. 5d) [61]. The plasma insulin concentration of diabetic rats also showed a continuously high level after oral administration. The combination of glucose-responsive materials and oral insulin delivery contributed to a safe and prolonged hypoglycemia effect.

The development of glucose-responsive oral insulin delivery has been limited due to the challenges involved in integrating glucose-responsive elements with oral delivery vehicles. Nevertheless, certain studies have made notable progress by incorporating glucose-responsive moieties with specifically designed materials for oral insulin delivery. These approaches have shown a promising paradigm for enhancing the regulation of blood glucose levels through oral insulin delivery. However, further research is needed to overcome the remaining obstacles and fully realize the potential of oral glucose-responsive insulin delivery.

## Conclusions and perspectives

Oral insulin delivery holds immense promise for the optimized management of diabetes, offering improved patient convenience and adherence. However, the aforementioned physiological barriers pose challenges to its successful implementation. In addition, variations in absorption among patients and the narrow therapeutic index of insulin further hinder its real-world applications.

Over the past few decades, extensive research has been conducted on delivery systems, aiming at protecting insulin and enhancing intestinal permeability, thereby significantly increasing its bioavailability (Table 1). The harsh gastrointestinal environment can be overcome through the use of acid-resistant materials, including synthetic polymeric materials, inorganic materials, and MOFs. Furthermore, zwitterionic and surface-modified carriers have demonstrated their ability to improve intestinal penetration and enhance bioavailability in animal models. To enhance oral bioavailability, it is crucial to continue advancing materials design. One approach is to integrate elements that provide both insulin protection and enhanced permeability. Achieving a delicate balance between mucus penetration and epithelial crossing or realizing both properties in a single material, would greatly facilitate the preparation of oral insulin delivery systems. In addition, the concept of oral glucose-responsive insulin delivery represents a next-generation system wherein glucose-responsive moieties are integrated with oral delivery materials, offering the potential for improved diabetes management with a reduced risk of hypoglycemia.

**Table 1:** Delivery systems, aiming at protecting insulin, enhancing intestinal permeability and realizing glucose-responsive insulin release

Objectives	Material designs	Mechanism or properties	Results	Ref.
Insulin protection in gastrointestinal tract	Polymerized ursodeoxycholic acid	Protonated and hydrophobic under acidic conditions	Minimal leakage of insulin in the acid conditions	[13]
	Amphiphilic dendrimer with benzoboroxole			[14]
	Hyaluronic acid-modification	Protection effects of hyaluronic acid shell	Less than 10% leakage of insulin in the acid conditions	[15]
	Hydroxypropyl-β-cyclodextrin	Hydrophobic central cavity and host-guest interactions with insulin	50.1% insulin left after one hour of α-chymotrypsin treatment	[16]
	Porous silicon nanoparticles	High physiochemical stability of silicon-based materials	Negligible insulin release at pH 1.2 and 6.5	[20]
	NU-1000	Acid-stable structures; Proper pore size for loading insulin and excluding enzymes	Minimal release of insulin in the stomach milieu	[22]
	UiO-68-NH_2_		Release more than 90% in PBS and less than 20% in the stomach	[23]
	MIL-100		Negligible insulin release at pH 5.4 and accelerated dramatically at pH 7.4	[24]
Permeability enhancement	DSPE-PCB	Diameter less than 100 nm; Zwitterionic surface; Ultra-low critical micelle concentration; Receptor-mediated absorption	Achieving about 42.6% bioavailability in diabetic rats	[33]
	PCB/insulin nanoparticles		Achieving about 27.0% bioavailability in diabetic rats	[34]
	Dilauroylphosphatidylcholine	Diameter about 90 nm; Zwitterionic surface; Receptor-mediated absorption	About 4.76% bioavailability in diabetic rats	[35]
	PLGA modified with R8 and phosphoserine	Diameter about 100 nm; Zwitterionic surface; Charge reversal by enzyme digestion	About 5.96% bioavailability in diabetic rats	[36]
	Carboxymethyl-β-cyclodextrin modified with R8	R8-mediated cellular transport	A three-fold enhanced transport efficiency after R8 modification on Caco-2 cells	[38]
	Insulin and penetrain complex coated with pHPMA	pHPMA coating contributing to good mucus penetration and gradual dissociating in mucus; Penetratin resulting high cellular uptake	20-fold higher absorption on the mucus-secreting cellular than free insulin	[41]
	Poly (lactic acid)-b-poly (ethylene glycol) modified with Fc	Diameter about 60 nm; Fc-mediated cellular transport	11.5-fold higher absorption efficiency in mice than without modification	[44]
	PLGA-PEG modified with albumin	Diameter about 150 nm; Albumin-mediated Fc receptor targeted cellular transport	2-fold higher transcytosis efficiency in polarized epithelial than without modification	[46]
	Polymeric nanoparticles modified with transferrin	Diameter about 100 nm; Transferrin-mediated cellular transport	5-fold higher cellular uptake in Caco-2 cells than modified with bovine serum albumin	[47]
Oral glucose-responsive release of insulin	Core-shell structure liposome reversely conjugated with PBA	PBA detaching when intestinal glucose increases; The exposed Fc facilitating cellular uptake	Effectively reduction of postprandial blood glucose excursions	[15]
	Nanoparticles composed of DSPE-PEG-Mal and DSPE-PEG-Mal and with Fc modification	Fc-mediated cellular transport; The PBA pinacol ester-mediated glucose-responsive insulin release	Achieving a superior hypoglycemic effect up to 16 h	[45]
	H_2_O_2_ sensitive polymer loaded with GOx	Polymer oxidation under hyperglycemic conditions, triggering rapid insulin release	Achieving hypoglycemic effect up to 12 h even after food challenge	[4]
	nCOF	Glucose replacing the space between nanosheets, triggering insulin release	Achieving hypoglycemic effect and high plasma insulin levels up to 10 h	[61]

However, clinical translation of oral insulin persists with tremendous challenges, as evidenced by the limited FDA-approved protein drugs and their relatively low bioavailability [62]. The lack of extensive experimental validation in large animals makes it difficult to accurately predict insulin’s efficacy in humans. Moreover, before considering clinical use, it is crucial to thoroughly investigate the potential long-term systemic toxicity of the materials employed in oral insulin delivery. These challenges highlight the need for further research and rigorous evaluation to address the concerns surrounding oral insulin delivery. Preclinical studies involving large animal models are necessary steps to establish the safety and efficacy of oral insulin delivery systems for human use. Only through comprehensive investigation and validation can validate the potential of oral insulin as a viable treatment option.

## Data Availability

Not applicable.

## References

[1] Sun H (2022). IDF diabetes atlas: global, regional and country-level diabetes prevalence estimates for 2021 and projections for 2045. Diabetes Res Clin Pract.

[2] Mathieu C (2021). One hundred years of insulin therapy. Nat Rev Endocrinol.

[3] Mitragotri S (2014). Overcoming the challenges in administering biopharmaceuticals: formulation and delivery strategies. Nat Rev Drug Discovery.

[4] Wang A (2020). Liver-target and glucose-responsive polymersomes toward mimicking endogenous insulin secretion with improved hepatic glucose utilization. Adv Func Mater.

[5] Chu J (2022). Foundations of gastrointestinal-based drug delivery and future developments. Nat Rev Gastroenterol Hepatol.

[6] Drucker DJ (2020). Advances in oral peptide therapeutics. Nat Rev Drug Discovery.

[7] Lock JY (2018). Mucus models to evaluate the diffusion of drugs and particles. Adv Drug Deliv Rev.

[8] Turner JR (2009). Intestinal mucosal barrier function in health and disease. Nat Rev Immunol.

[9] Xiao Y (2020). Oral insulin delivery platforms: strategies to address the biological barriers. Angew Chem Int Ed.

[10] Brown TD (2020). Materials for oral delivery of proteins and peptides. Nat Rev Mater.

[11] Yang Y (2023). Recent advances in oral and transdermal protein delivery systems. Angew Chem Int Ed.

[12] Mo R (2014). Emerging micro- and nanotechnology based synthetic approaches for insulin delivery. Chem Soc Rev.

[13] Lee JS (2021). Metabolic and immunomodulatory control of type 1 diabetes via orally delivered bile-acid-polymer nanocarriers of insulin or rapamycin. Nat Biomed Eng.

[14] Zeng Z (2019). Stimuli-responsive self-assembled dendrimers for oral protein delivery. J Control Release.

[15] Yu J (2019). Glucose-responsive oral insulin delivery for postprandial glycemic regulation. Nano Res.

[16] Li S (2021). Inclusion complex based on N-acetyl-L-cysteine and arginine modified hydroxypropyl-β-cyclodextrin for oral insulin delivery. Carbohydr Polym.

[17] Asal HA (2022). Controlled synthesis of in-situ gold nanoparticles onto chitosan functionalized PLGA nanoparticles for oral insulin delivery. Int J Biol Macromol.

[18] He Y (2020). Protective properties of mesocellular silica foams against aggregation and enzymatic hydrolysis of loaded proteins for oral protein delivery. J Colloid Interface Sci.

[19] Hristov D (2020). Silica-coated nanoparticles with a core of zinc, l-arginine, and a peptide designed for oral delivery. ACS Appl Mater Interfaces.

[20] Martins JP (2022). Neonatal Fc receptor-targeted lignin-encapsulated porous silicon nanoparticles for enhanced cellular interactions and insulin permeation across the intestinal epithelium. Bioact Mater.

[21] Katsoulidis AP (2019). Chemical control of structure and guest uptake by a conformationally mobile porous material. Nature.

[22] Chen Y (2018). Acid-resistant mesoporous metal–organic framework toward oral insulin delivery: protein encapsulation, protection, and release. J Am Chem Soc.

[23] Zou J (2022). Efficient oral insulin delivery enabled by transferrin-coated acid-resistant metal-organic framework nanoparticles. Sci Adv.

[24] Zhou Y (2020). A nanocomposite vehicle based on metal–organic framework nanoparticle incorporated biodegradable microspheres for enhanced oral insulin delivery. ACS Appl Mater Interfaces.

[25] Nielsen L (2001). Probing the mechanism of insulin fibril formation with insulin mutants. Biochemistry.

[26] Hubálek F (2020). Molecular engineering of safe and efficacious oral basal insulin. Nat Commun.

[27] Kjeldsen TB (2021). Engineering of orally available, ultralong-acting insulin analogues: discovery of OI338 and OI320. J Med Chem.

[28] Wu L (2018). Engineering nanomaterials to overcome the mucosal barrier by modulating surface properties. Adv Drug Deliv Rev.

[29] Griesser J (2018). Self-emulsifying peptide drug delivery systems: How to make them highly mucus permeating. Int J Pharm.

[30] Verma A (2010). Effect of surface properties on nanoparticle–cell interactions. Small.

[31] Li Q (2022). Zwitterionic biomaterials. Chem Rev.

[32] Cone RA (2009). Barrier properties of mucus. Adv Drug Deliv Rev.

[33] Han X (2020). Zwitterionic micelles efficiently deliver oral insulin without opening tight junctions. Nat Nanotechnol.

[34] Li Y (2021). Charge-switchable zwitterionic polycarboxybetaine particle as an intestinal permeation enhancer for efficient oral insulin delivery. Theranostics.

[35] Shan W (2016). Enhanced oral delivery of protein drugs using zwitterion-functionalized nanoparticles to overcome both the diffusion and absorption barriers. ACS Appl Mater Interfaces.

[36] Wu J (2018). Biomimetic viruslike and charge reversible nanoparticles to sequentially overcome mucus and epithelial barriers for oral insulin delivery. ACS Appl Mater Interfaces.

[37] Zorko M (2005). Cell-penetrating peptides: mechanism and kinetics of cargo delivery. Adv Drug Deliv Rev.

[38] Yang L (2018). A cell-penetrating peptide conjugated carboxymethyl-β-cyclodextrin to improve intestinal absorption of insulin. Int J Biol Macromol.

[39] Niu Z (2018). PEG-PGA enveloped octaarginine-peptide nanocomplexes: An oral peptide delivery strategy. J Control Release.

[40] Khafagy E (2012). Oral biodrug delivery using cell-penetrating peptide. Adv Drug Deliv Rev.

[41] Shan W (2015). Overcoming the diffusion barrier of mucus and absorption barrier of epithelium by self-assembled nanoparticles for oral delivery of insulin. ACS Nano.

[42] Florence AT (2012). “Targeting” nanoparticles: the constraints of physical laws and physical barriers. J Control Release.

[43] Sockolosky JT (2015). The neonatal Fc receptor, FcRn, as a target for drug delivery and therapy. Adv Drug Deliv Rev.

[44] Pridgen EM (2013). Transepithelial transport of Fc-targeted nanoparticles by the neonatal Fc receptor for oral delivery. Sci Transl Med.

[45] Xiao Y (2021). Glucose-responsive oral insulin delivery platform for one treatment a day in diabetes. Matter.

[46] Azevedo C (2020). Engineered albumin-functionalized nanoparticles for improved FcRn binding enhance oral delivery of insulin. J Control Release.

[47] Zhu X (2016). Polymeric nanoparticles amenable to simultaneous installation of exterior targeting and interior therapeutic proteins. Angew Chem Int Ed.

[48] Xi Z (2022). Dual-modified nanoparticles overcome sequential absorption barriers for oral insulin delivery. J Control Release.

[49] Zhang X (2014). Biotinylated liposomes as potential carriers for the oral delivery of insulin. Nanomedicine.

[50] Ke Z (2015). Efficient peroral delivery of insulin via vitamin B12 modified trimethyl chitosan nanoparticles. J Pharm Pharm Sci.

[51] Zheng Y (2018). Multifunctional nanoparticles enable efficient oral delivery of biomacromolecules via improving payload stability and regulating the transcytosis pathway. ACS Appl Mater Interfaces.

[52] Yu J (2016). Stimuli-responsive delivery of therapeutics for diabetes treatment. Bioeng Transl Med.

[53] Liu W (2022). Macroencapsulation devices for cell therapy. Engineering.

[54] Bakh NA (2017). Glucose-responsive insulin by molecular and physical design. Nat Chem.

[55] Wang Z (2021). Developing insulin delivery devices with glucose responsiveness. Trends Pharmacol Sci.

[56] Yao Y (2022). Materials and carriers development for glucose-responsive insulin. Acc Mater Res.

[57] Wang J (2020). Glucose-responsive insulin and delivery systems: innovation and translation. Adv Mater.

[58] Yu J (2015). Microneedle-array patches loaded with hypoxia-sensitive vesicles provide fast glucose-responsive insulin delivery. Proc Natl Acad Sci.

[59] Wang J (2019). Glucose transporter inhibitor-conjugated insulin mitigates hypoglycemia. Proc Natl Acad Sci.

[60] Yu J (2020). Glucose-responsive insulin patch for the regulation of blood glucose in mice and minipigs. Nat Biomed Eng.

[61] Benyettou F (2021). In vivo oral insulin delivery via covalent organic frameworks. Chem Sci.

[62] Cao S (2019). Nanoparticles: oral delivery for protein and peptide drugs. AAPS PharmSciTech.

